# Emergency department visits for children identified as at risk of mental and behavioral conditions in the United States: an analysis of the 2019 NHIS data

**DOI:** 10.1186/s12913-025-12792-9

**Published:** 2025-05-02

**Authors:** Samuel N. Koscelny, David M. Neyens, Ann Dietrich, Danielle Stewart, Veronica Parker, Anjali Joseph

**Affiliations:** 1 Department of Industrial Engineering, Clemson University, 100 Freeman Hall, Clemson, SC 29631 USA; 2 Department of Bioengineering, Clemson University, Clemson, SC USA; 3 Prisma Health System, Greenville, SC USA; 4 School of Nursing, Clemson University, Clemson, SC USA; 5 School of Architecture, Clemson University, Clemson, SC USA

**Keywords:** Pediatric, Mental health, Survey data, Emergency departments, Strengths and Difficulties Questionnaire (SDQ)

## Abstract

**Background:**

The prevalence of mental and behavioral health (MBH) conditions in children has been increasing in the past two decades. Emergency departments (EDs) are also experiencing a significant rise in MBH-related visits, leading to challenges in providing care. Gaining insight into the underlying characteristics of pediatric patients at higher risk of MBH conditions is crucial for understanding this population in the ED and addressing their complex care needs. This study aims to examine the characteristics of children reported to be at risk and not at risk of MBH conditions to identify the population characteristics associated with ED visits. The objective was to analyze data from the 2019 National Health Interview Survey (NHIS) to evaluate the odds of ED visits among children and to identify patterns among those at higher risk of MBH conditions.

**Methods:**

The study utilized data from the 2019 NHIS Sample Child Survey, focusing on children aged 6–17. Following established guidelines, children with a Strengths and Difficulties Questionnaire total score of 16 or higher were classified as having higher risk of MBH conditions. Binary logistic regression and ordinal logistic regression analyses were conducted in R. Three models were created; the first two examined factors among the general pediatric population associated with one ED visit or multiple ED visits within a year. The last model examined only children at higher risk of MBH conditions and the factors associated with ED visits in this sub-population.

**Results:**

The weighted sample size of the survey consisted of 49,330,998 children. Approximately 15.8% of children had been to the ED at least once in the past year and 6.6% of children were at risk of MBH conditions. The regression analyses revealed children reported at higher risk of MBH conditions were significantly more likely to visit the ED. Other factors associated with ED visits included preexisting health conditions such as asthma, suboptimal health status, and financial strain. Among children at higher risk of MBH conditions, having a consistent primary care setting (e.g., doctor’s office or health center) was associated with significantly lower odds of visiting the ED.

**Conclusions:**

The study provides insights into the characteristics of children with and without risk of MBH conditions, as well as their associated odds of ED visits. Understanding these factors can contribute to interventions and improvements within the ED for children presenting for MBH-related conditions. Further research is needed to improve care for this patient population in the ED.

## Introduction

In 2021, the American Academy of Pediatrics, the American Academy of Child and Adolescent Psychiatry, and the Children’s Hospital Association collectively declared a national emergency in children’s mental health in the United States [3]. The prevalence of mental and behavioral health (MBH) challenges in children and young adults has increased in recent years [32]. In the U.S., data from 2022 revealed that 15% of youth experienced a major depressive episode [32], and of the youth with major depression, over 60% did not receive mental health treatment [32]. Along with the rise of MBH conditions in the youth population, emergency departments (EDs) have experienced a profound increase in MBH-related visits in the past two decades [10, 21, 23, 26, 34, 38], with visits doubling from 2011 to 2020 [9].

In light of the rising number of MBH‐related visits, it is critical to recognize how these presentations differ from medical or trauma‐related visits. EDs serve as a crucial safety net for children at risk for MBH challenges [9] and encounter patients with a broad range of diagnoses–including depression, anxiety, agitation and aggression, self-harm behavior, suicidal ideation, and substance use and abuse [27]. There is a particularly notable rise observed among youth with autism spectrum disorder (ASD), who access ED services four times more often than youth without ASD and require additional care needs [15, 25, 44]. Multiple factors may contribute to increased ED utilization, including psychiatric inpatient bed shortages, private and public insurance changes, and a shortage of pediatric-trained mental health specialists in the community [14]. MBH-related visits often require different resources from those provided for medical or trauma patients [14], and EDs may lack the necessary support to effectively address these unique needs [27]. Consequently, this often results in extended wait times that can be distressing to children with certain MBH needs and increase the risk that the pediatric patient and their family would abandon seeking care at all [13, 18, 37]. Understanding these unique demands underscores the urgency of addressing MBH‐related challenges in ED settings and offers insight into the broader factors influencing pediatric ED utilization.

Research has shown that both patients with MBH conditions and the clinicians who care for them often report negative experiences, likely stemming from inadequate resources, limited training, and insufficient support within the emergency department setting [31, 45]. This suggests the ED system may need additional support for clinicians and other health professionals in managing pediatric patients with MBH conditions. One way to support this effort is to understand more about the children who receive care in the ED – an approach that is well framed by the Andersen and Aday Behavioral Model of Health Services Use. A comprehensive examination of demographic, socioeconomic, and health-related characteristics can guide the development of targeted, data-driven interventions to optimize care and resource allocation [1, 4–6].

Past research—whether explicitly or implicitly guided by the Andersen and Aday Behavioral Model—has leveraged statistical models to deepen our understanding of patient populations across diverse healthcare settings [2]. For specific MBH-related purposes, studies have used electronic health record (EHR) data to identify early warning signs and risk factors for depression in young adults [30] and have analyzed national survey data to identify factors associated with ED visits among adults with MBH indicators [8]. However, most existing research has centered on the pediatric population who has presented to the ED with MBH-related concerns, highlighting rising visit rates, prolonged lengths of stay, and persistent disparities in care [19, 27]. In contrast, less is known about pediatric populations at higher risk of MBH conditions who may present to the ED for chief complaints that are not related to mental or behavioral health.

Our study addresses this gap by utilizing the National Health Interview Survey (NHIS) data [29] to examine the underlying characteristics of general pediatric ED utilization—with a particular focus on youth identified as at higher risk of MBH conditions compared to those who are not. In our analysis, “children at higher risk of MBH conditions” is defined using the Strengths and Difficulties Questionnaire (SDQ), a validated screening tool that the NHIS uniquely implemented in 2019 to gather comprehensive national mental health data among children. This approach enables us to identify children at higher MBH risk and to explore how a range of demographic, socioeconomic, and health-related factors influence overall ED use—even when individual visits are not explicitly classified as MBH-related. In this study, we have two main objectives: (1) to identify determinants associated with ED utilization among all children, and (2) to examine how these factors may differ among children at higher risk of MBH conditions.

## Methods

### Data source: National Health Interview survey

The National Health Interview Survey (NHIS), administered by the National Center for Health Statistics, is designed to collect information on a wide range of health-related topics, such as health status, healthcare access and utilization, health behaviors, and health conditions [29]. To ensure representativeness of the population of the United States, the collected data is weighted [29]. The survey’s topics and inquiries have evolved over time, therefore the data collected each year is subject to annual variation. The NHIS survey includes questions for both adults and children within a household. The Sample Child component of the NHIS focuses on children under 18 years old with detailed questions about the child’s health, development, and well-being. Parents or guardians complete the questionnaire on behalf of their children,youth and adolescents do not self-complete the survey. For this study we only used the data from 2019 Sample Child Survey because it included the Strengths and Difficulties Questionnaire (SDQ components. We included children in the developmental periods of middle childhood (ages 6 to 9 years, preadolescent phase (ages 10 to 13 years, and adolescence (ages 14 to 17 years old similar to previous work investigating this pediatric population [43]. For each variable we have noted the specific survey question identifiers parenthetically in the following sections.

### Outcome measures

This study focused on the factors associated with at least one ED visit within the prior 12 months preceding the survey (EMERG12MTC_C). From this variable, two outcome variables were derived: one binary measure and one ordinal measure. The binary variable was coded as (1, child visited an ED at least one time in the previous 12 months; 0; they had not). The ordinal variable was coded as (1, child visited the ED one time in the previous 12 months; 2, child visited the ED two times in the previous 12 months; 3, child visited the ED three times in the previous 12 months; 4, child visited the ED four or more times in the previous 12 months; and 0, they had not visited the ED).

### Explanatory measures

#### Theoretical justification for variable selection

Our choice of explanatory measures was guided by the Andersen and Aday Behavioral Model of Health Services Use, which posits that health service utilization is influenced by a combination of predisposing, enabling, and need factors [1, 4–6]. In line with this framework, we selected variables that capture a range of demographic, socioeconomic, and health-related characteristics. Specifically, we included measures reflecting children’s age, sex, insurance status, family economic context, as well as indicators of ASD diagnosis and risk of MBH conditions, as these factors are consistently recognized in health services research as key determinants of care-seeking behavior.

#### Classification of children at higher risk of MBH conditions

To define children at higher risk of MBH conditions in our study population, we employed the SDQ component of the NHIS – recognizing the SDQ’s established reliability and utility for large scale epidemiological studies [29]. Additionally, its inclusion in the 2019 NHIS was unique, as the SDQ is part of this national survey on a rotating basis. In the NHIS dataset, the SDQ total score (SDQTOT_C) is a continuous variable ranging from 0 to 40, derived from four subscales: emotional symptoms, conduct problems, hyperactivity/inattention, and peer relationship problems. While the SDQ also assesses a fifth subscale, prosocial behavior, it is scored separately, as it measures positive social behaviors rather than difficulties [29]. For this study, we identified children at higher risk of MBH conditions if their score was 16 or higher on the SDQ, a cutoff informed by its validated categories to include those in both the upper threshold of the borderline and in the abnormal ranges. As noted by Vugteveen et al. [42], the SDQ is not a diagnostic instrument but rather provides a preliminary indication of potential MBH concerns. Thus, by adopting a sensitive threshold, our approach ensures that we capture children who fall within the upper borderline range as well as those scoring in the abnormal range, aligning with our study’s goal of using the SDQ to identify children at higher risk of MBH conditions and its associations with emergency department utilization.

#### Coding methodology

After identifying the relevant factors, we recoded each variable as binary indicators to facilitate clear interpretation in our multivariable models. Demographic variables such as sex (SEX_C) and age (AGEP_C) were binarily categorized to align with research conventions and to capture potentially distinct usage patterns among subgroups (e.g., younger children versus older adolescents, male versus female). In addition to standard demographic measures, we included variables reflecting clinical need. Children diagnosed with ASD (ASDEV_C), which includes clinical diagnoses of ASD or pervasive developmental disorder, were incorporated into our model. The presence of this diagnosis was recoded as (1, reported with ASD diagnosis), while the absence of these conditions were recoded as (0, not reported with ASD diagnosis). Children at higher risk of MBH conditions were recoded as (1, child is at higher risk of MBH conditions; 0, child is not at higher risk of MBH conditions).

Health-related characteristics were also systematically coded. The child’s overall health (PHSTAT_C) was recoded to distinguish between those reporting “good, “fair”, or “poor” health (coded as 1) and those reporting “very good” or “excellent” health (coded as 0), including “preferred not to answer” and nonresponses in the latter category. We defined our cutoff to include all children with suboptimal perceived health—those reporting “good,” “fair,” or “poor” health—in contrast to children with optimal health (“very good” or “excellent”), ensuring a clear distinction between these groups. We also included preexisting health conditions such as if a child was reported as having diabetes (1, child has diabetes; 0, child does not have diabetes; DIBEV_C) and if the child was reported as having asthma (1, child has asthma; 0, does not have asthma; ASEV_C).

Socioeconomic factors were represented by several measures. Food security (FDSCAT4_C) was recoded as (1, low food security; 0, not low food security). We dichotomized the child’s usual care setting by (1, child’s usual care setting is a doctor’s office or health center; 0, child’s usual care setting is not a doctor’s office or health center; USPLKIND_C). Additional factors such as the child’s family reported difficulty paying bills (1, child’s family reported difficulty paying bills; 0, family did not report difficulty paying bills; PAYBLL12M_C) and if the child’s family had private health insurance (1, family had private health insurance; 0, family did not have private health insurance; COVER_C) were recoded and included in the analysis.

Taken together, these variables reflect the Andersen and Aday model’s tripartite structure—predisposing (e.g., age, sex), enabling (e.g., insurance status, family economic factors), and need (e.g., poor, fair, or good health, risk of MBH conditions, chronic conditions)—allowing us to assess their combined impact on pediatric ED utilization in a comprehensive, theory-driven manner.

### Statistical analysis

The data was analyzed in R (version 4.2.2) and the Survey package (version 4.1–1) was utilized to account for the weights within the data. To understand the underlying characteristics associated with ED visits within the prior year, we created three models. The first model we constructed was a binary logistic regression model to determine significant characteristics of children visiting the ED at least once, incorporating data for both children with and without higher risk of MBH conditions. Following this, we created an ordinal logistic regression model to examine the factors associated with the varying number of visits to the ED among all the children in the dataset. To focus on the unique characteristics of children at higher risk of MBH conditions, we developed a second binary logistic regression model using data exclusively from this subgroup. This focused approach allowed for a more granular epidemiological insight into the distinct factors driving ED utilization among children at higher risk of MBH conditions.

For both our general and MBH-specific binary logistic regression models, the outcome variable was defined as whether a child visited the ED at least once in the past 12 months. In these analyses, we employed the *svyglm* function from the Survey package. Using the *svyolr* function for the ordinal logistic regression model, we recoded the dependent variable as an ordinal measure to represent the different frequencies of a child’s ED visits in the past year. As the weighted sample size was large and represented a population of nearly fifty-million children in the first two models, $\alpha$ = 0.001 was used for our population level models to assess significance. In the model that included only children at higher risk of MBH conditions, a significant level of $\alpha$ = 0.05 was used because of the smaller sample size.

## Results

### Descriptive statistics

The NHIS dataset included 6,301 children aged 6–17 years before applying survey weights. After applying the survey weights, the sample size of children in the dataset was 49,330,998, reflecting the estimated population in the United States in 2019. The survey data indicated that approximately 24,186,840 (49.0%) children were identified as female. The average age of the children of respondents was 11.95 years old (SD = 3.47) and 34.1% of the reported family income was over $50,000. Nearly 27.5 million children (57.7%) were reported to have private health insurance. Most of the children (92.0%, approximately 45.4 million), were reported to have a primary care setting at a doctor’s office, while about 4 million (8.0%) did not. An estimate of 7,800,955 (15.8%) children were reported to have been to the emergency department at least once in the last twelve months. Out of the total number of children in the survey, 1,475,257 (3.0%) were reported as having ASD, while 3,252,731 (6.6%) were reported to have higher risk of MBH conditions. These descriptive statistics can be seen in Table 1. Table 5 in the Appendix presents the distribution of ED visit frequencies (0, 1, 2, 3, and 4 + visits) across all explanatory variables included in the statistical models, with row percentages for each variable category.

**Table 1:** Characteristics of children between 6 years and 17 years old; N, weighted = 49,330,998

Characteristic	Participants, weighted, n (%)	
Age	Mean=11.95, SD=3.47	
Sex		
Male	25,144,158	(51.0)
Female	24,186,840	(49.0)
Financial Difficulties		
Family reported difficulty paying bills	7,090,402	(14.4)
Family did not report difficulty paying bills	42,240,596	(85.6)
Health status		
Reported as either poor, fair, or good	6,642,385	(13.5)
Not reported as either poor, fair, or good	42,688,613	(86.5)
Asthma status		
Child has asthma	6,595,082	(13.4)
Child does not have asthma	42,735,916	(86.6)
Insurance status		
Child covered by private health insurance	27,472,949	(57.7)
Child not covered by private health insurance	20,159,299	(42.3)
Primary care setting		
Doctor’s office or health center	45,360,457	(92.0)
Not a doctor’s office or health center	3,970,541	(8.0)
Emergency Department (ED) Visits		
Been to the ED at least once in last year	7,800,955	(15.8)
Has not been to the ED in last year	41,530,043	(84.2)
ASD status		
Child has ASD diagnosis	1,475,257	(3.0)
Child does not have ASD diagnosis	47,855,741	(97.0)
Mental and behavioral health status		
Child is at higher risk of MBH conditions	3,252,731	(6.6)
Child is not at higher risk of MBH conditions	46,078,267	(93.4)

### Factors associated with at least 1 ED visit within the prior year among all children

A binary logistic regression model was used to examine the factors associated with whether children had at least one ED visit (Table 2). Among pediatric patients, those diagnosed with asthma (adjusted odds ratio (AOR) = 1.67, 99% CI 1.26 to 2.21) had higher odds of visiting the ED. Children from families that reported difficulty paying bills in the past 12 months (AOR = 1.45, 99% CI 1.10 to 1.92) or had low food security (AOR = 1.51, 99% CI 1.14 to 1.99) also had significantly increased odds of an ED visit. By contrast, children with private insurance coverage had lower odds of ED utilization (AOR = 0.67, 99% CI 0.53 to 0.86). Furthermore, children whose overall health was classified as poor, fair, or good had higher odds to visit the ED (AOR = 1.66, 99% CI 1.20 to 2.29). Children at higher risk of MBH conditions had 1.66 times higher odds to visit the ED (99% CI 1.12 to 2.46).

**Table 2:** Logistic regression model to predict factors associated with at least 1 ED visit within the prior year among all children

Factor	Estimate	SE	t-value	*P* value	Odds ratio (99% CI)
(Intercept)	−1.82	0.21	−8.76	< 0.001	0.16 (0.10, 0.28)
Child age is younger than 12 years old	<.01	0.10	0.00	0.999	1.00 (0.78, 1.28)
Sex is male	−0.10	0.09	−1.03	0.30	0.91 (0.72, 1.15)
Health is classified as poor, fair, or good	0.51	0.13	4.04	< 0.001	1.66 (1.20, 2.29)
Covered by private insurance	−0.40	0.09	−4.23	< 0.001	0.67 (0.53, 0.86)
Family reported difficulty paying bills in last 12 months	0.37	0.11	3.46	< 0.001	1.45 (1.10, 1.92)
Child has asthma	0.51	0.11	4.68	< 0.001	1.67 (1.26, 2.21)
Child has diabetes	1.41	0.44	3.23	0.001	4.10 (1.33, 12.64)
Child has ASD diagnosis	0.03	0.24	0.14	0.892	1.03 (0.55, 1.94)
Child lived with an individual with substance use disorder	0.23	0.13	1.79	0.07	1.26 (0.90, 1.76)
Child has low food security	0.41	0.11	3.83	< 0.001	1.51 (1.14, 1.99)
Child’s primary care setting is doctor’s office/health center	<.01	0.21	0.00	0.999	1.00 (0.59, 1.71)
Child is at higher risk of MBH conditions	0.51	0.15	3.31	< 0.001	1.66 (1.12, 2.46)
Model statistics parameters					
AIC			5096.5		
Dispersion parameter			1.00		

### Factors associated with multiple ED visits within the prior year among all children

An ordinal logistic regression model was employed to assess factors associated with multiple ED visits during the past year (Table 3). The intercept terms (e.g., “Intercept: no visit | 1 visit”) mark the cutoff points between categories of ED visit frequency and are not interpreted as predictors. Children whose overall health status was reported as poor, fair, or good were more likely to have multiple ED visits (AOR = 1.70, 99% CI 1.23 to 2.36). Children with private insurance had a lower odds of multiple ED visits (AOR = 0.67, 99% CI 0.53 to 0.85). In contrast, children from families experiencing financial difficulties (AOR = 1.43, 99% CI 1.08 to 1.90) or low food security (AOR = 1.51, 99% CI 1.15 to 1.99), as well as those with asthma (AOR = 1.69, 99% CI 1.28 to 2.24), had increased odds of visiting the ED multiple times. Furthermore, children identified as being at higher risk of MBH conditions had 1.72 times higher odds to have repeated ED visits (99% CI 1.15 to 2.58).

**Table 3:** Ordinal logistic regression model to predict factors associated with multiple ED visits within the prior year among all children

Factor	Estimate	S.E.	t-test	*P*-value	Odds ratio (99% CI)
Intercept: no visit | 1 visit	1.82	0.2	8.88	< 0.001	6.14 (3.63, 10.40)
Intercept: 1 visit | 2 visits	2.99	0.21	14.06	< 0.001	19.82 (11.46, 34.25)
Intercept: 2 visits | 3 visits	4.38	0.23	18.93	< 0.001	79.93 (44.04, 145.08)
Intercept: 3 visits | 4 visits	5.04	0.27	18.66	< 0.001	154.98 (77.27, 310.86)
Child age is younger than 12 years old	0.01	0.1	0.15	0.88	1.01 (0.79, 1.30)
Sex is male	−0.08	0.09	−0.91	0.36	0.92 (0.72, 1.17)
Health is classified as poor, fair, or good	0.53	0.13	4.17	< 0.001	1.70 (1.23, 2.36)
Covered by private insurance	−0.4	0.09	−4.36	< 0.001	0.67 (0.53, 0.85)
Family reported difficulty paying bills in last 12 months	0.36	0.11	3.32	< 0.001	1.43 (1.08, 1.90)
Child has asthma	0.52	0.11	4.81	< 0.001	1.69 (1.28, 2.24)
Child has diabetes	1.14	0.37	3.09	0.002	3.12 (1.21, 8.02)
Child has ASD diagnosis	<.01	0.25	0	1	1.00 (0.53, 1.88)
Child lived with an individual with substance use disorder	0.23	0.13	1.73	0.08	1.26 (0.89, 1.77)
Child has low food security	0.41	0.11	3.88	< 0.001	1.51 (1.15, 1.99)
Child’s primary care setting is doctor’s office/health center	−0.01	0.21	−0.06	0.95	0.99 (0.58, 1.69)
Child is at higher risk of MBH conditions	0.54	0.16	3.48	< 0.001	1.72 (1.15, 2.58)
Model statistics parameters					
Deviance	6879.81				

### Factors associated with at least 1 ED visit among children at higher risks of MBH conditions within the prior year

For children at higher risk of MBH conditions, a binary logistic regression model was developed to predict the factors associated with having at least one ED visit in the past 12 months (Table 4). Given the smaller population sample of children at higher risk of MBH conditions or being identified with ASD, we used $\alpha$ = 0.05 to assess significance in this model. Results indicate that children with asthma had higher odds of visiting the ED (AOR = 2.23, 95% CI 1.05 to 4.74). Children experiencing low food security were also at increased odds (AOR = 2.21, 95% CI 1.05 to 4.63), while male children had lower odds to visit the ED (AOR = 0.43, 95% CI 0.19 to 0.98). Additionally, having a usual place for care was associated with lower odds of an ED visit (AOR = 0.22, 95% CI 0.05 to 0.90).

**Table 4:** Logistic regression model to predict factors associated with at least one ED visit among children at higher risks of MBH conditions within the prior year

Factor	Estimate	SE	t-value	*P* value	Odds ratio (95% CI)
(Intercept)	0.39	0.60	0.65	0.52	1.48 (0.31, 7.02)
Child age is younger than 12 years old	−0.12	0.28	−0.44	0.66	0.89 (0.43, 1.81)
Sex is male	−0.83	0.31	−2.65	0.01	0.43 (0.19, 0.98)
Health is classified as poor, fair, or good	0.12	0.28	0.43	0.67	1.13 (0.55, 2.33)
Covered by private insurance	−0.17	0.33	−0.52	0.60	0.84 (0.36, 1.96)
Family reported difficulty paying bills in last 12 months	0.40	0.26	1.52	0.13	1.49 (0.76, 2.93)
Child has asthma	0.80	0.29	2.74	0.01	2.23 (1.05, 4.74)
Child has diabetes	1.54	1.42	1.08	0.28	4.66 (0.12, 181.86)
Child has ASD diagnosis	−0.06	0.35	−0.16	0.87	0.94 (0.39, 2.31)
Child lived with an individual with substance use disorder	0.24	0.28	0.87	0.39	1.27 (0.62, 2.58)
Child has low food security	0.79	0.29	2.75	0.01	2.21 (1.05, 4.63)
Child’s primary care setting is doctor’s office/health center	−1.51	0.54	−2.77	0.01	0.22 (0.05, 0.90)
Model statistics parameters					
AIC	480.91				
Dispersion parameter	1.05				

## Discussion

This study identifies factors associated with ED utilization among all children and provides unique insight into the subpopulation of children at higher risk of MBH conditions, illustrating how their ED utilization patterns compare to the broader pediatric population. Although our study focuses on children at higher risk of MBH conditions rather than exclusively on specific MBH‐related ED visits, the widespread increase in MBH-related visits to EDs [32] underscores the importance of leveraging national data to identify population characteristics and understand ED utilization patterns of children at higher risks for MBH conditions. Our models showed that children at higher risk of MBH conditions were significantly more likely to present to the ED. Additional factors, including insurance status, preexisting health conditions, and financial difficulties, were also significantly associated with ED utilization among all U.S. children.

In our analysis, we included covariates that may impact a child’s odds of presenting to the ED, aligning with the Andersen and Aday framework. This approach considers how healthcare utilization is influenced by predisposing factors (e.g., demographic characteristics), enabling factors (e.g., access to care), and need factors (e.g., health status). Through this lens, our study examines how these covariates interact to shape patterns of ED use among pediatric populations. For example, while prior research demonstrates that conditions such as asthma and diabetes are prevalent among pediatric populations [11, 28], with some studies highlighting mental health comorbidities in children with diabetes [36], the relationship between these conditions and ED utilization among youth at higher risk of MBH conditions remains an area requiring further exploration. Although diabetes did not reach statistical significance in our models, it was very close, suggesting that future studies might explore potential confounders or use alternative modeling strategies to better understand this association. Our findings indicate pediatric patients with other comorbidities have higher odds of ED visits. Specifically, in the overall U.S. population, we found that children with reported poor, fair, or good health had increased odds of at least one ED visit and multiple ED visits–a pattern not observed among children at higher risk of MBH conditions. Similarly, children with asthma had higher odds of at least one ED visit in both the overall U.S. sample and the MBH subgroup, and asthma was also linked to higher odds of multiple ED visits within the overall population.

Furthermore, insurance status also played a role, as privately insured children displayed lower odds of visiting the ED at least once or multiple times compared to uninsured or publicly insured children, echoing prior studies showing that the lack of private insurance is correlated with increased ED utilization [16, 40]. Hoge et al. [22] and Bommersbach et al. [9] also found that health insurance coverage among children with MBH conditions, in particular, those with public health insurance, may be associated with higher odds of presenting to the ED. Our models indicate that financial difficulties and food insecurity–which are forms of material hardship [33]–were significantly associated with increased odds of ED visits among all children. Prior research has demonstrated material hardship’s role as a critical measure for economic vulnerability [35] and is significantly correlated with higher ED visit probabilities [17]. However, in the model focusing on children at higher risk of MBH conditions, only food insecurity remained a significant predictor. Given that past research which has found material hardship to be a precise predictor of adverse mental health outcomes [7, 39], future studies should further explore the mechanisms linking financial instability to ED utilization and assess targeted interventions to mitigate these risks. Overall, our results are consistent with the findings of previous literature regarding the link between a child’s health condition, insurance status, and financial challenges and ED utilization patterns.

In the models representing all children, our findings indicate that children at higher risk of MBH conditions exhibit significantly higher odds for at least one ED visit and for multiple visits. This aligns with prior literature showing children with MBH conditions are outpacing those without MBH conditions in ED utilization [9, 21, 23]. It is important to note that our models did not differentiate between ED visits specifically related to MBH concerns (e.g., depression, suicidal ideation, aggressive behavior) and visits for other medical conditions (e.g., asthma, infections). Children at higher risk of MBH conditions may utilize the ED for a wide range of reasons, many of which may be unrelated to their MBH status. However, all children at higher risk of MBH conditions may benefit from tailored support services, such as mental health specialists, to address their unique needs during a healthcare encounter. Notably, while our overall models of the U.S. pediatric population (Tables 2 and 3) found that having a doctor’s office or health center as the usual place of care was not significantly associated with the odds of any ED visit or multiple ED visits, our subpopulation analysis of children at higher risk of MBH conditions (Table 4) revealed this factor to be significantly associated with lower odds of ED use. This finding is consistent with prior research showing that children who lack regular, continuous care—often due to not consistently seeing the same primary care provider—are more likely to rely on the ED than those with established, ongoing care relationships [12, 20, 22]. Future research should explore utilization patterns among children at higher risk of MBH conditions—specifically examining whether subsets of this population predominantly seek care for MBH concerns, while others use the ED for non-MBH medical needs. Such research could elucidate the role that consistent, continuous care plays in improving care for this pediatric subpopulation.

Taken together, these findings illustrate the link between low continuity of care and higher overall ED utilization, as children at higher risk of MBH conditions are more likely to have multiple ED visits (Table 3). While previous research underscores the value of equipping EDs with dedicated resources and trained healthcare specialists to effectively address pediatric MBH needs [9, 41], our results also reinforce the theoretical value of early detection and proactive intervention–a position similarly supported by Koning et al. [24]. Within the ED context itself, the Andersen and Aday model [1, 4–6] offers a framework for improving care experiences for children with or at higher risk of MBH conditions. Such strategies could include sensory accommodations for children with ASD and structured protocols tailored to pediatric MBH patients or those at higher risks. In this way, early identification complements enhanced in-ED support: while emergency departments must be equipped to address the immediate needs of children at elevated risk for MBH conditions, appropriate outpatient follow-up and access to community-based resources are essential to support the ongoing needs of these children—whether their emergency visit was due to MBH concerns or other medical issues. Collectively, these strategies may enhance the quality of care for youth with higher risk of MBH conditions across healthcare settings.

### Limitations and future work

There are several limitations to be considered for the generalizability and implications of this study. We identified children at higher risk of MBH conditions based on the SDQ scores, which, as a screening tool, only indicates higher risk of underlying mental or behavioral health difficulties and may not reflect actual MBH diagnoses or conditions. This measure of risk of MBH conditions reflects a wide range of psychosocial challenges that may overlap with or be distinct from conditions such as ASD. Additionally, the NHIS data does not indicate whether an ED visit was related to MBH or other non–MBH-related concerns. As our results show, children at higher risk of MBH conditions are more likely to visit the ED at least once and to have multiple visits. It is worth noting that our models did not identify a significant association between ASD and the odds of ED utilization compared to children without ASD–a finding that may be influenced by the broader classification of MBH risk based on SDQ scores. In this manner, the ‘higher risk of MBH conditions’ variable may encompass a wide spectrum of psychosocial difficulties that not only overlap with ASD symptoms but also contribute to ED utilization independently of an ASD diagnosis. Nonetheless, as past research has found significantly higher ED utilization among children with ASD [25], future work should investigate the overlap of autism and other psychosocial factors contributing to ED utilization. Although race and ethnicity indicators are available in the NHIS dataset and may capture additional unmeasured influences on pediatric care pathways, we ultimately excluded these variables from the final analysis. In the dataset, the impact of race was largely mediated by other variables and other enabling characteristics,including race as a predictor risked redundancy and potential confounding.

Furthermore, our results show that children at higher risk of MBH conditions–a identified through a validated screening tool rather than a formal diagnosis–are more likely to visit the ED at least once and are more likely to have multiple visits. This brings attention to a key contribution of this work: by analyzing survey data such as the NHIS, which can include parent- or self-reported mental health indicators (in our study, it was the parent-reported SDQ), we can identify a broader group of at-risk children who may otherwise be unknown to have higher risk of MBH conditions and be overlooked in EHR clinical records. Previous studies have often focused on ED utilization using EHR data which has classified visits as a MBH pediatric visit or not a MBH pediatric visit. This has important implications not only for ED clinicians and administrators, but also for primary care providers, health system and health insurance administrators, and public health practitioners. Specifically, early identification of children at higher risk of MBH conditions could facilitate timely and appropriate referrals to MBH-specific care. Future work should explore integrated, multi-sectoral strategies—involving primary care, community services, and public health systems—to implement proactive interventions that bridge ED care with community-based support services. The NHIS data used in this analysis was collected before the COVID- 19 pandemic, so future analysis should evaluate how ED utilization patterns among children at elevated risk of MBH conditions may have changed after the pandemic.

## Conclusions

Using the NHIS 2019 data, this study examined factors affecting ED utilization, specifically focusing on children at higher risk of MBH conditions. Although the increasing prevalence of MBH conditions in children has led to a greater demand for ED services related to mental and behavioral health [10, 21, 23, 26, 34, 38], our findings indicate that children who are at higher risk of MBH conditions–regardless of formal diagnosis– are also significantly more likely to have higher overall ED utilization. While this trend poses challenges for the healthcare system, it also offers a valuable opportunity to innovate care models, enhance resource allocation, and ultimately improve outcomes for this population. As the results of this study show, factors such as preexisting health conditions, insurance status, and financial difficulties are significant factors which increase a child’s odds to visit the ED. Developing a comprehensive understanding of the characteristics of children who frequently use ED services can inform targeted interventions and innovative care strategies. In particular, leveraging predictors such as elevated SDQ scores can help identify children at higher risk of MBH conditions, enabling timely outpatient support and preventative care that extends beyond the emergency department setting.

## Data Availability

The datasets generated and/or analyzed during the current study are available in the 2019 NHIS data repository at https://www.cdc.gov/nchs/nhis/index.htm.

## Appendix

**Table 5:** Distribution of ED visit frequency (row percentages) for each binary coded explanatory variable

Variable	0 Visit (%)	1 Visit (%)	2 Visit (%)	3 Visit (%)	+4 Visit (%)
Child age is younger than 12 years old	84.3	9.7	4.6	0.6	0.8
Child age is 12 years old or older	83.8	10.6	3.9	0.9	0.8
Sex is male	84.3	10.0	4.2	0.8	0.7
Sex if female	83.8	10.3	4.3	0.7	0.9
Health is classified as poor, fair, or good	71.7	16.8	6.2	2.5	2.8
Health is not classified as poor, fair, or good	86.0	9.1	4.0	0.4	0.5
Covered by private insurance	87.9	8.0	3.2	0.5	0.4
Not covered by private insurance (including public insurance and no insurance)	78.6	13.2	5.7	1.1	1.4
Family reported difficulty paying bills in last 12 months	75.2	16.1	5.3	1.4	2.0
Family did not report difficulty paying bills in last 12 months	85.5	9.2	4.1	0.6	0.6
Child has asthma	74.1	16.0	5.5	1.6	2.8
Child does not have asthma	85.6	9.2	4.1	0.6	0.5
Child has diabetes	46.6	36.3	9.0	3.9	4.2
Child does not have diabetes	84.1	10.1	4.3	0.7	0.8
Child has ASD diagnosis	76.7	15.0	3.8	1.9	2.6
Child does not have ASD diagnosis	84.2	10.0	4.3	0.7	0.8
Child lived with an individual with substance use disorder	76.8	14.3	5.5	0.6	2.8
Child did not live with an individual with substance use disorder	84.9	9.7	4.1	0.7	0.6
Child has low food security	73.9	16.0	6.4	1.9	1.8
Child does not have low food security	86.3	8.8	3.8	0.5	0.6
Child’s primary care setting is doctor’s office/health center	84.1	10.1	4.2	0.8	0.8
Child’s primary care setting is not a doctor’s office/health center	81.8	11.2	5.1	0.2	1.7
Child is at higher risk of MBH conditions	68.6	15.8	8.2	3.2	4.2
Child is not at higher risk of MBH conditions	85.0	9.8	4.0	0.6	0.6

## Abbreviations

MBH: Mental and behavioral health
ED: Emergency department
ASD: Autism spectrum disorder
EHR: Electronic health record
NHIS: National Health Interview Survey
SDQ: Strengths and Difficulties Questionnaire
